# An acute phase protein α_1_-acid glycoprotein mitigates AKI and its progression to CKD through its anti-inflammatory action

**DOI:** 10.1038/s41598-021-87217-8

**Published:** 2021-04-12

**Authors:** Hiroshi Watanabe, Rui Fujimura, Yuto Hiramoto, Ryota Murata, Kento Nishida, Jing Bi, Tadashi Imafuku, Hisakazu Komori, Hitoshi Maeda, Ayumi Mukunoki, Toru Takeo, Naomi Nakagata, Motoko Tanaka, Kazutaka Matsushita, Masafumi Fukagawa, Toru Maruyama

**Affiliations:** 1 Department of Biopharmaceutics, Graduate School of Pharmaceutical Sciences, Kumamoto University, 5-1 Oe-Honmachi, Chuo-ku, Kumamoto, 862-0973 Japan; 2 Program for Leading Graduate Schools “HIGO (Health Life Science: Interdisciplinary and Glocal Oriented) Program”, Kumamoto University, 5-1 Oe-Honmachi, Chuo-ku, Kumamoto, 862-0973 Japan; 3 Division of Reproductive Engineering, Center for Animal Resources and Development (CARD), Kumamoto University, Kumamoto, Japan; 4 Department of Nephrology, Akebono Clinic, 1-1 Shirafuji 5 Chome, Minami-ku, Kumamoto, 861-4112 Japan; 5 Division of Nephrology, Endocrinology and Metabolism, Tokai University School of Medicine, 143 Shimo-Kasuya, Isehara, 259-1193 Japan

**Keywords:** Acute kidney injury, Chronic kidney disease

## Abstract

The molecular mechanism for acute kidney injury (AKI) and its progression to chronic kidney disease (CKD) continues to be unclear. In this study, we investigated the pathophysiological role of the acute phase protein α_1_-acid glycoprotein (AGP) in AKI and its progression to CKD using AGP KO mice. Plasma AGP levels in WT mice were increased by about 3.5-fold on day 1–2 after renal ischemia–reperfusion (IR), and these values then gradually decreased to the level before renal IR on day 7–14. On day 1 after renal IR, the AGP KO showed higher renal dysfunction, tubular injury and renal inflammation as compared with WT. On day 14, renal function, tubular injury and renal inflammation in WT had recovered, but the recovery was delayed, and renal fibrosis continued to progress in AGP KO. These results obtained from AGP KO were rescued by the administration of human-derived AGP (hAGP) simultaneously with renal IR. In vitro experiments using RAW264.7 cells showed hAGP treatment suppressed the LPS-induced macrophage inflammatory response. These data suggest that endogenously induced AGP in early renal IR functions as a renoprotective molecule via its anti-inflammatory action. Thus, AGP represents a potential target molecule for therapeutic development in AKI and its progression CKD.

## Introduction

Acute kidney injury (AKI) was generally thought to be a reversible condition with a favorable prognosis for the complete recovery of renal function, but recent epidemiological studies have shown that AKI is associated with increased risk of mortality and other adverse outcomes, and that the severity or frequency of AKI is intimately related to a poor outcome^1–11^. In addition, AKI is an important risk factor for chronic kidney disease (CKD) and end-stage renal disease (ESRD), a pathway to CKD that is referred to as an “AKI to CKD transition”. In fact, a systematic review and meta-analysis demonstrated that, in the patients with AKI, the pooled incidence of CKD and ESRD were 25.8 per 100 person-years and 8.6 per 100 person-years, respectively^12^. Based on these findings, the onset of AKI and its progression to CKD have become a clinical issue because no effective treatments are currently available.

Regarding the mechanism responsible for the progression from AKI to CKD, it has been reported that incomplete renal repair “maladaptive repair” after renal tubule injury could be involved^13–17^. A normal renal repair “adaptive repair” occurs if the tubular injury is mild. Under such conditions, the dead cells of the renal tubule are removed by living tubular epithelial cells and infiltrated macrophages. Thereafter, the proliferation of surviving tubular epithelial cells compensates for the missing cells due to cell death, thus completing tubular regeneration. The infiltrated macrophages then change their polarity into anti-inflammatory M2-type macrophages, converge on the inflammation and promote tubular regeneration. On the other hand, in the case of “maladaptive repair”, it is generally thought that the renal repair process becomes abnormal due to the prolonged inflammation, which eventually results in the development of renal fibrosis, which is a common finding of tubular atrophy and CKD progression^15–17^. The prolonged inflammation associated with this “maladaptive repair” could be important in the progression of AKI to CKD, but there are still many unclear points regarding the cause of the prolonged inflammation. Therefore, in order to suppress the progression of AKI to CKD, it is essential to understand the inflammatory response in the progression of AKI to CKD.

α_1_-acid glycoprotein/orosomucoid (AGP/ORM), a serum protein with a molecular weight of approximately 45 kDa, is composed of 183 amino acids and five *N*-linked sugar chains that account for approximately 45% of the molecular weight of the molecule^18,19^. AGP is mainly produced in the liver, but is also produced by monocytes and alveolar macrophages^20^. AGP is known as an acute phase protein whose expression increases during conditions of inflammation, and its plasma concentration increases by 2–5 times during inflammation^21,22^. It is also known that AGP has physiological functions such as anti-inflammatory and immunoregulatory functions. In fact, we previously reported that AGP up-regulates CD163 expression in macrophages, thereby reversing their polarity and converting them into anti-inflammatory macrophages^23^. We also recently reported that a unilateral ureteral obstruction in mice resulted in increased endogenous plasma AGP levels, and that systemic AGP knockout (KO) mice were more likely to develop renal fibrosis than wild-type (WT) mice^24^. These data indicate that endogenous AGP may have a renal protective function. In fact, exogenous administration of human derived-AGP (hAGP) to unilateral ureteral obstruction-induced renal fibrotic mice and to adriamycin-induced nephropathy mice suppressed renal fibrosis and proteinuria by changing macrophages polarization into M2-like anti-inflammatory macrophages in the kidney^25,26^.

Based on the above background, we hypothesized that the acute phase protein AGP has a protective function in the inflammatory response during AKI and its progression to CKD via its anti-inflammatory action. To confirm this hypothesis, we examined (1) the time course for plasma AGP levels in AKI to CKD model mice and (2) the involvement of AGP in the onset of AKI and its progression to CKD using AGP KO mouse.

## Results

### Plasma AGP level is increased after renal ischemia–reperfusion in AKI to CKD mice

**Figure 1:**
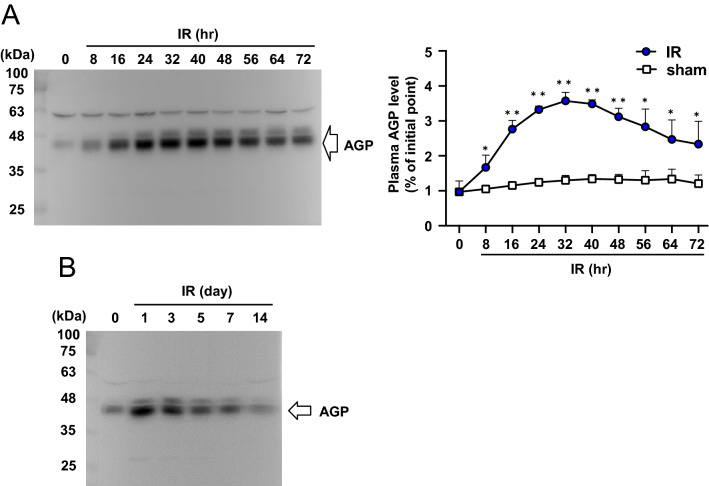
Plasma AGP level is increased after renal IR. Plasma AGP levels in mice before IR (0 h) and post IR (8 to 72 h), and (**B**) before IR (day 0) and post IR (day 1 to day 14) were analyzed by Western blotting using anti-AGP antibody. Data are expressed as means ± SE (n = 3). *P < 0.05, **P < 0.01 compared with sham mice at each time point.

AKI to CKD mice were prepared by ischemia–reperfusion (IR) of both renal arteries and veins for 35 min, as described in a previous report^27^. The change in plasma AGP level after the renal IR treatment in wild type (WT) mice was first examined by Western blotting. As a result, the plasma AGP level was increased by up to approximately 3.5 times at 24–48 h after renal IR (day 1–2), and then gradually decreased (Fig. 1A). At 14 days after the renal IR, the plasma AGP level returned to the level previous to the IR (Fig. 1B).

### Systemic AGP knockout exacerbates renal function and renal inflammation at day 1 after renal ischemia–reperfusion

**Figure 2:**
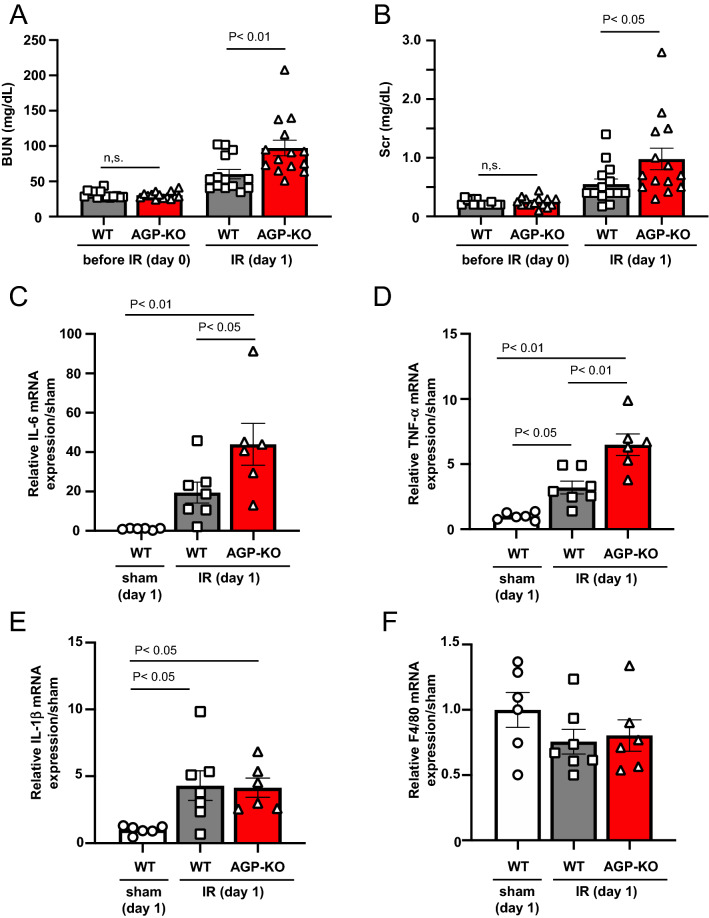
Systemic AGP knockout exacerbates renal function and renal inflammation at day 1 after renal ischemia–reperfusion. (**A**) Blood urea nitrogen (BUN) and (**B**) serum creatine (SCr) were measured at day 1 after renal IR (n = 14). The mRNA expression of (**C**) IL-6, (**D**) TNF-α, (**E**) IL-1β and (**F**) F4/80 in kidney at day 1 after renal IR were determined by real-time PCR (n = 6–7). Data are expressed as means ± SE.

To clarify the pathophysiological significance of the increased AGP after renal IR, we conducted a study using systemic AGP KO mice. In the WT and AGP KO mice before IR (day 0), there were no significant differences in renal function (BUN, SCr) (Fig. 2A,B), body weight (Supplemental Fig. 1A), the expression of Kim-1 mRNA, a renal tubular injury marker (Supplemental Fig. 1B) and renal histological evaluation (PAS staining) (Supplemental Fig. 1C). At 1 day after the renal IR (day 1), although no significant difference in body weight was observed between the WT (IR) and AGP KO (IR) mice (Supplemental Fig. 1A), the AGP KO (IR) mice showed an increase in BUN, SCr and Kim-1 mRNA expression as compared to WT (IR) (Fig. 2A,B and Supplemental Fig. 1B). Consistent with these results, the histological evaluation by PAS staining also confirmed that AGP KO (IR) tended to show increased cast formation and tubular injury compared to WT (IR) (Supplemental Fig. 1C). These data indicate that renal function and tissue damage after renal IR was exacerbated in the case of the AGP KO (IR) mice compared to the WT (IR) mice.

Renal inflammation after renal IR was also evaluated. At 1 day after renal IR (day 1), renal IL-6 and TNF-α mRNA expression were significantly increased in the AGP KO (IR) mice compared to the WT (IR) mice (Fig. 2C,D), while no significant changes were observed in IL-1β and F4/80 mRNA expression, a macrophage marker, between the AGP KO (IR) and WT (IR) mice (Fig. 2E,F). These results indicate that at 1 day after the renal IR, the kidney of the AGP KO (IR) mice were in a pro-inflammatory state compared with that of the WT (IR) mice. These data indicate that the IR-induced renal injury was exacerbated in the AGP KO (IR) mice compared to the WT (IR) mice, and that the inflammatory response could have contributed to the differences in the renal pathology between the AGP KO (IR) and WT (IR) mice. These findings suggest that endogenous AGP exerts a renal protective effect against AKI through its anti-inflammatory properties.

### Systemic AGP knockout exacerbates AKI to CKD progression

**Figure 3:**
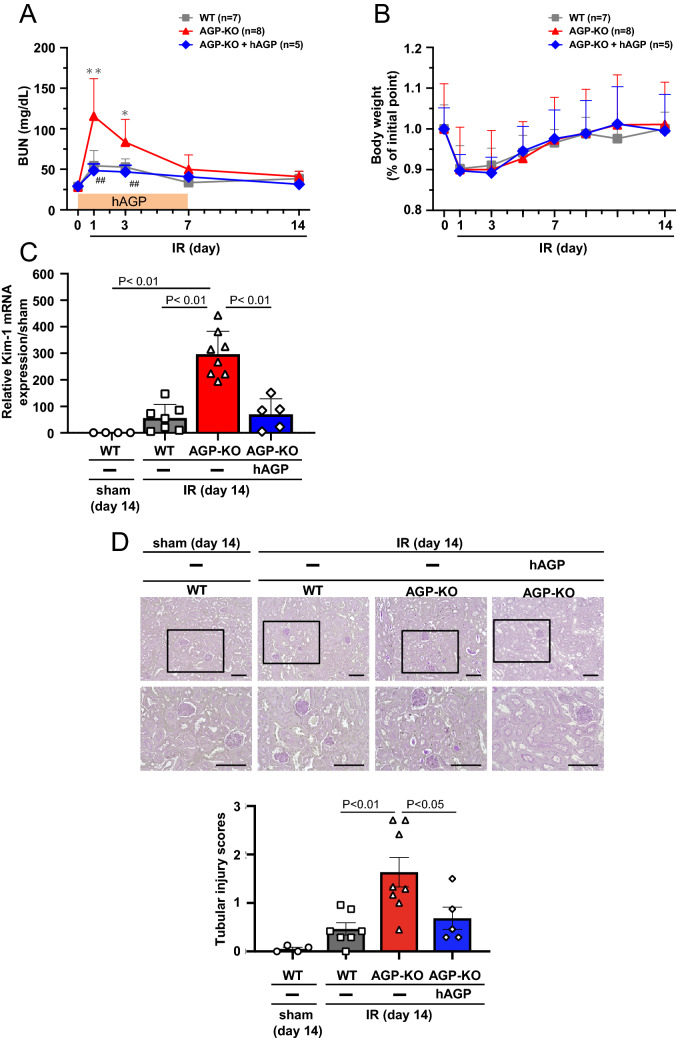
Systemic AGP knockout exacerbates AKI to CKD progression. (**A**) Blood urea nitrogen (BUN) and (**B**) body weights were measured up to day 14 after renal IR. hAGP administration (2 mg/mouse/day, *ip*, from day 0 (before IR) to day 7) to AGP KO mice suppressed IR-induced the increase of BUN. Date are expressed as means ± SE. *P < 0.05, **P < 0.01 compared with WT mice at each time point. ^#^P < 0.05, ^##^P < 0.01 compared with AGP KO mice at each time point. (**C**) Kim-1 mRNA expression in the kidney at day 14 after renal IR was determined by real-time PCR. (**D**) Representative photomicrographs of PAS-stained kidney sections at day 14 after renal IR are shown. Lower panels of PAS are an enlarged image of the upper panel. Original magnification: × 200 (upper panels), × 400 (lower panels). Scale bars represent 100 μm. hAGP administration to AGP KO mice rescued IR-induced renal injury. Data are expressed as the means ± SE (n = 4–8).

In order to examine the pathophysiological role of endogenous AGP in the progression of AKI to CKD, an evaluation was performed at 14 days after the renal IR treatment (day 14). In addition, as shown in Fig. 1, since plasma AGP levels were increased immediately after the renal IR in the WT mice and this higher AGP level was sustained for around 7 days, the rescue effect of exogenously administered human-derived AGP (hAGP) to AGP KO (IR) (2 mg/mouse/day, *ip*, from day 0 (before IR) to day 7) was also investigated. A significant increase in BUN was observed on day 1 and day 3 after renal IR in the AGP KO (IR) mice compared to the WT (IR) mice, and the administration of hAGP to the AGP KO (IR) mice suppressed this increase in BUN (Fig. 3A). On day 14, BUN, SCr, and body weight were not significantly different among the WT (IR), AGP KO (IR) and AGP KO (IR) + hAGP groups (Fig. 3A,B and Supplemental Fig. 2). On the other hand, Kim-1 mRNA levels at day 14 were significantly increased in the AGP KO (IR) mice compared to the sham or the WT (IR) mice (Fig. 3C). In addition, a renal histological evaluation using PAS staining indicated that a higher renal tissue injury had occurred in the AGP KO (IR) mice compared to the WT (IR) mice (Fig. 3D). At this time, the administration of hAGP (from day 0 (before IR) to day 7) to AGP KO (IR) significantly suppressed an increase in Kim-1 mRNA expression and the changes in renal histology (Fig. 3C,D).

### Administration of hAGP rescues renal fibrosis and inflammation at day 14 after renal-ischemia reperfusion in AGP KO mice

**Figure 4:**
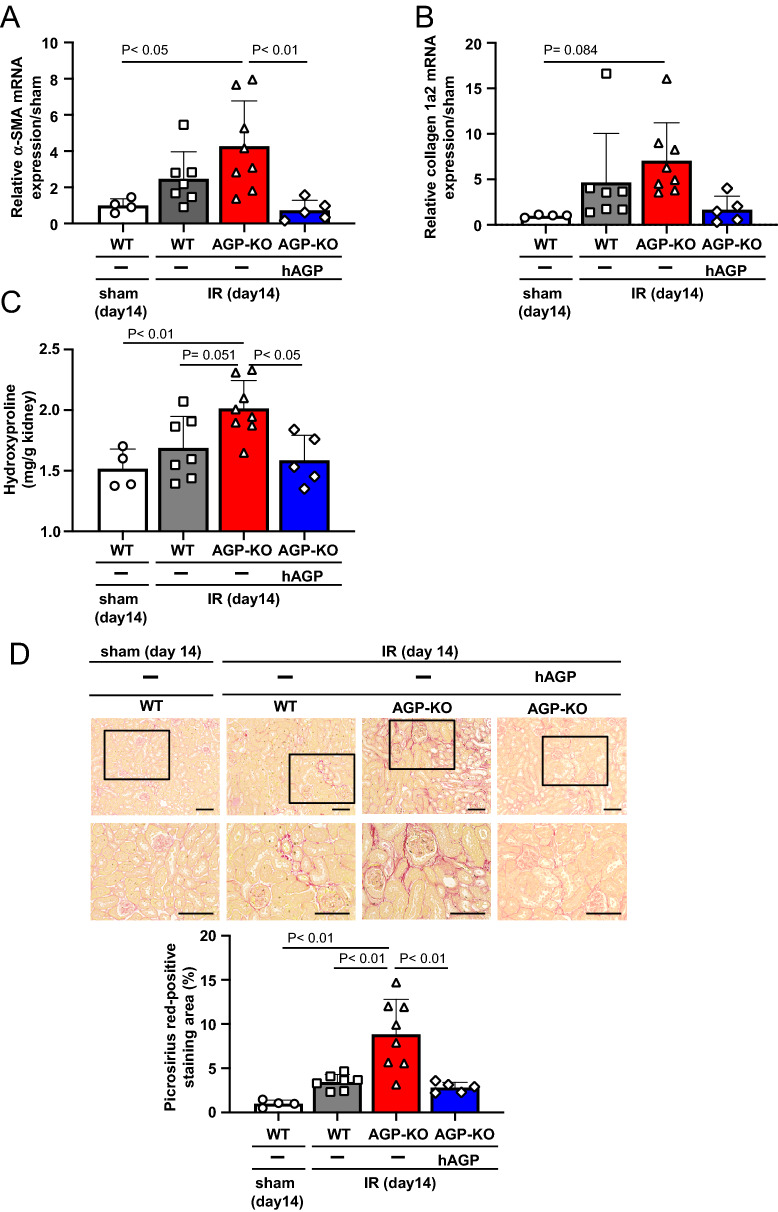
Administration of hAGP rescues renal fibrosis at day 14 after renal-IR in AGP KO mice. mRNA expression of (**A**) α-SMA and (**B**) Collagen 1a2 in kidney at day 14 after renal IR were determined by real-time PCR. (**C**) Hydroxyproline levels in the kidney were measured at 14 days after renal IR. (**D**) Representative photomicrographs of Picrosirius red-stained kidney sections and a semi-quantitative scoring analysis of picrosirius red-positive staining area at 14 after renal IR are shown. Lower panels are an enlarged image of the upper panel. Original magnification: × 200 (upper panels), × 400 (lower panels). Scale bars represent 100 μm. hAGP administration to AGP KO mice (2 mg/mouse/day, *ip*, from day 0 (before IR) to day 7) rescued IR-induced renal fibrosis. Date are expressed as the means ± SE (n = 4–8).

A study of renal fibrosis was conducted on day 14. Renal mRNA expression levels of α-SMA and collagen 1a2, myofibroblast markers, were evaluated. As a result, the expression levels of α-SMA and collagen 1a2 mRNA tended to increase in the AGP KO (IR) mice compared to the WT (IR) mice (Fig. 4A,B). Similarly, hydroxyproline levels were also increased in the AGP KO (IR) mice compared to the WT (IR) mice (Fig. 4C). Picrosirius red staining showed that AGP KO (IR) mice significantly increased renal fibrosis area as compared to WT mice (Fig. 4D). The administration of hAGP (from day 0 (before IR) to day 7) to AGP KO (IR) mice significantly suppressed the extent of renal fibrosis that was observed in the AGP KO (IR) mice (Fig. 4A–D).

**Figure 5:**
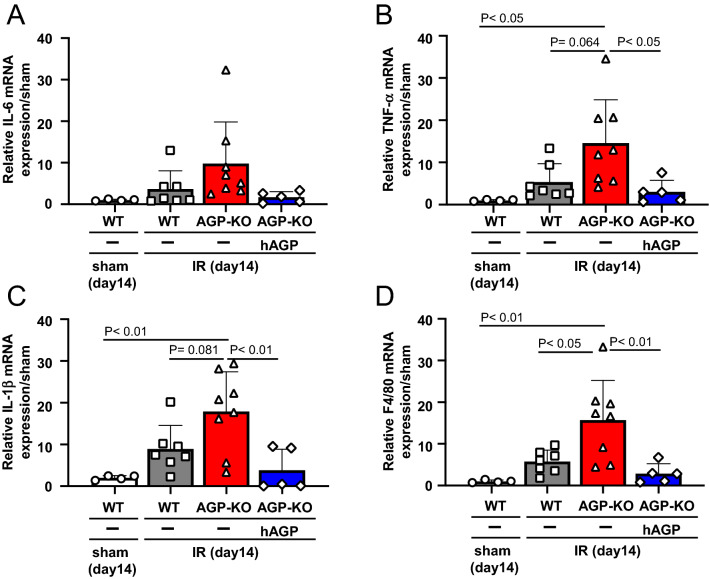
Administration of hAGP rescues renal inflammation at day 14 after renal-IR in AGP KO mice. The mRNA expression of (**A**) IL-6, (**B**) TNF-α, (**C**) IL-1β and (**D**) F4/80 in the kidney at day 14 after renal IR were determined by real-time PCR. hAGP administration to AGP KO mice (2 mg/mouse/day, *ip*, from day 0 (before IR) to day 7) suppressed IR-induced renal inflammation. Data are expressed as the means ± SE (n = 4–8).

The extent of renal inflammation was also examined on day 14. As a result, renal IL-6, TNF-α and IL-1β mRNA expression tended to increase in the AGP KO (IR) mice compared to the WT (IR) mice (Fig. 5A–C). Regarding F4/80, as shown in Fig. 2, no increase in F4/80 was observed on day 1, but a significant increase was observed on day 14 in the AGP KO (IR) mice compared to the WT (IR) mice (Fig. 5D). Under the same experimental conditions, the administration of hAGP (from day 0 (before IR) to day 7) to AGP KO (IR) suppressed the elevation of IL-6, IL-1β, TNF-α and F4/80 that was observed in the AGP KO (IR) mice. From these results, we conclude that the AGP KO (IR) mice had an enhanced renal inflammatory response and renal fibrosis on day 14 compared to the WT (I/R) mice and, as a result, the exogenous administration of hAGP suppressed them.

### hAGP ameliorates a lipopolysaccharide-induced macrophage inflammatory response

**Figure 6:**
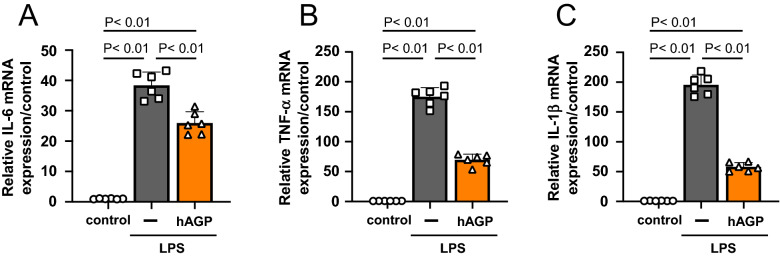
hAGP ameliorates lipopolysaccharide-induced macrophage inflammatory response. RAW 264.7 were exposed to 1.0 mg/mL D-PBS or hAGP for 48 h, and then exposed to 100 ng/mL LPS in the absence of hAGP for 3 h. mRNA expression of (**A**) IL-6, (**B**) TNF-α and (**C**) IL-1β in RAW 264.7 were determined by real-time PCR. Data are expressed as the mean ± SE (n = 6).

In vivo data using AGP KO mice suggested that endogenous AGP functions as a renal protective molecule against the renal IR-induced transition of AKI to CKD through its anti-inflammatory action. To confirm this, the effect of AGP on the inflammatory response of macrophages was examined using an in vitro experimental system. In this experiment, mouse macrophage cells (RAW 264.7) were incubated in the presence or absence of AGP, and the inflammatory response on LPS stimulation was evaluated. As a result, LPS stimulation caused a significant increase in IL-6, TNF-α and IL-1β mRNA expression, whereas a pretreatment with hAGP (1.0 mg/mL: plasma AGP level as observed during inflammation) significantly suppressed this LPS-induced inflammatory response (Fig. 6A–C). From these results, we conclude that AGP functions in a protective manner against the inflammatory response on mouse macrophages.

## Discussion

In recent years, it has been demonstrated that the severity of AKI is deeply related to the prognosis of patients, and that AKI is a risk factor for the development of CKD. However, there are many unclear points that remain regarding the molecular mechanism of AKI onset and its progression to CKD. In this study, we investigated the pathophysiological role of the acute phase protein AGP in renal IR-induced AKI and its progression to CKD using AGP KO mice, and found that endogenous AGP functions as a renoprotective molecule via its anti-inflammatory action.

Daemen et al. and de Vries et al. et al. previously reported that a single administration of exogenous hAGP (administered just before IR or immediately after IR) exerted a renal protective effect using renal IR-induced AKI mice^28,29^. Specifically, the exogenous administration of hAGP was found to ameliorate the renal dysfunction, neutrophil infiltration, inflammation and apoptosis after a renal IR treatment. On the other hand, de Vries et al. also showed that renal IR-induced AKI was not prevented in transgenic mice overexpressing rat AGP. These conflicting results suggest that a transient increase in the plasma AGP concentration after renal IR may play an important role as a renal protective effect against AKI. However, the pathophysiological role of endogenous AGP in AKI remains unclear at this time. Therefore, in this study, we evaluated the role of endogenous AGP in renal IR-induced AKI and its progression to CKD using AGP KO mice. First, in WT mice, plasma AGP levels were increased by about 3.5-fold at 24 to 48 h after the renal IR treatment (day 1–2). This value then gradually decreased to the level before renal IR on day 7–14 (Fig. 1A). Compared to WT (IR) mice, AGP KO (IR) mice showed a further increase in BUN and SCr at day 1 (Fig. 2A,B), and Kim-1 expression and tubular injury tended to increase at day 1 (Supplemental Fig. 1B and C). It is generally believed that renal damage could be resolved if the renal repair normally occurs after AKI. In fact, Kim-1 expression and the tubular injury score in WT (IR) mice were recovered on day 14 compared to day 1 (Fig. 3 and Supplemental Fig. 1). On the other hand, in AGP KO (IR) mice, the recovery of Kim-1 expression and tubular injury scores were delayed compared with those for WT (IR) mice. Furthermore, on day 14, renal fibrosis was progressing in AGP KO (IR) mice compared with WT (IR) mice, although the similar renal functions (BUN and SCr) for the WT (KO) and AGP-KO (IR) mice were observed. These results obtained from AGP KO (IR) mice were rescued by the administration of hAGP (from day 0 (before IR) to day 7). Therefore, endogenously induced AGP in early renal IR could be involved in the protection against the onset of AKI and its progression to CKD. A similar anti-fibrotic action of endogenous AGP was also observed in unilateral ureteral ligation renal fibrosis mice^24^.

We further post-administered hAGP to AGP KO mice after renal IR to investigate the effect of AGP on the transition of AKI to CKD. hAGP was administered to the AGP KO mice from 1 day after renal IR (from day 1 to day 7) (Figure S3). However, the data showed that the post-administration of hAGP failed to suppress renal inflammation and fibrosis at day 14. These data demonstrate that early endogenous AGP induction until 1 day after renal IR could contribute to the suppression of AKI and its progression to CKD. In other words, the more severe AKI observed in the AGP KO compared to WT mice (Fig. 3A) could have led to the development of a more severe fibrosis. As shown in Figure S3, an increase of BUN in AGP KO (IR) (Figure S3) was higher than that shown in Fig. 3A despite the same ischemic time. Therefore, to confirm the contribution of endogenous or exogenously administered AGP on the transition of AKI to CKD, adjusting the severity of AKI would be needed. In addition, a dose escalation effect of post-administering hAGP would be needed in the future.

As shown in Figs. 2 and 5, we evaluated the inflammatory response after renal IR treatment. As a result, the kidneys on day 1 and day 14 after renal IR were in a pro-inflammatory state, and at any evaluation point, a higher inflammation was observed in the AGP KO (IR) mice compared to the WT (IR) mice. In renal IR models, macrophages are involved in all phases of the injury process, including the early injury, subsequent repair and late fibrosis. Especially, proinflammatory macrophages contribute to the early injury phase^30–32^. Regarding the anti-inflammatory effect of AGP, we have previously found that AGP weakly activates the TLR4/CD14 signaling then increases the CD163 expression in macrophages^23^. Alvarado-Vazquez et al. reported that the CD163-overexpressing macrophages inhibit LPS-induced inflammation in vitro^33^. Furuhashi et al. also found that CD163-expressing macrophages are associated with IL-10 production as an anti-inflammatory cytokine in anti-GBM glomerulonephritis rat^34^. These findings indicated that AGP potentiates the suppression of renal inflammation by changing the macrophage phenotype to one with an enhanced CD163 expression via TLR4/CD14 signaling. In fact, as shown in Fig. 6, the hAGP treatment suppressed the LPS-induced macrophage inflammatory response in mouse-derived macrophages as well. The macrophages that produce pro-inflammatory cytokines also produce fibrosis-promoting factors such as TGF-β and connective tissue growth factor^35^ which also contribute to the progression of renal fibrosis. Therefore, it is possible that an increase in inflammation in the early renal IR contributed to the prolonged inflammation and renal fibrosis, as was observed in the AGP KO (IR) mice. These data indicate that endogenous AGP could have an anti-inflammatory effect by preventing inflammation derived from macrophages.

Our data using AGP KO mice indicate that endogenous AGP plays an important role in suppressing renal damage during the IR-induced AKI and its progression to CKD. Moreover, the rescue experiment by hAGP that mimics endogenous AGP induction after renal IR indicates that an early transient increase in endogenous AGP functions as a renoprotective factor because the transient administration of hAGP prevented disease progression. To verify this, it would be necessary to conduct a clinical study directed at determining whether the initial plasma AGP levels at AKI or its induction potency by AKI is associated with renal prognosis after AKI. If this is correct, AGP would be a predictive marker for assessing the severity of the transition of AKI to CKD.

In this study, we used systemic AGP KO mice. The main source of AGP production is the liver, but some AGP is also produced by monocytes and macrophages^20^. Therefore, future studies using monocyte- or macrophage-specific AGP KO mice would be necessary to determine whether the AGP produced by monocytes or macrophages is responsible for the anti-inflammatory effects of the AGP reported in this study.

## Conclusion

We report herein that endogenously induced-AGP not only contributes to the suppression of renal dysfunction and renal tissue damage after renal IR but also contributes to the suppression of renal fibrosis by functioning in a protective manner against the inflammatory response. These data provide a pathophysiological role for endogenous AGP in the onset of AKI and its progression to CKD. Thus, AGP represents a potential target molecule for therapeutic development in AKI or its progression to CKD, and AGP or AGP-inducing agents could be a potential therapeutic agent against AKI and its progression to CKD.

## Methods

### AKI to CKD model induced by renal ischemia–reperfusion (IR)

The study was carried out in compliance with the ARRIVE guidelines. The Animal Care and Use Committee of Kumamoto University approved the protocols for all animal experiments. All animals were housed in an environment with a controlled temperature, a 12 h light/dark cycle and with free access to food and water. AGP KO mice were generated by the Institute of Resource Development and Analysis Center for Animal Resources and Development (CARD), Kumamoto University, Japan^24^. Four-week-old male C57BL/6NcrSlc obtained from Japan SLC, Inc (Shizuoka, Japan) and 4-week-old male AGP KO mice were bred and used at the age of eight weeks. AKI to CKD model mice were induced by bilateral renal ischemia for 35 min. Body weights were measured at days 1, 3, 5, 7, 9, 11, 14 after renal IR. Mice were sacrificed at day 1 and day 14 after renal IR. All methods were performed in accordance with the relevant guidelines and regulations, and were approved by Kumamoto University.

### Renal function

Blood samples were collected at day 0, 1, 3, 7, and 14 after renal IR. BUN and serum creatinine were measured using FUJI DRI-CHEM (Tokyo, Japan).

### Histological analysis

After perfusion with saline, a part of the kidney was incubated with 4% formaldehyde for 24 h at 4 °C and embedded in paraffin. Sections of renal tissue (2 μm) were stained with Periodic acid-Schiff (PAS) and Picrosirius red. Images were obtained using a Keyence BZ-X710 microscope (Osaka, Japan). Tubular injury in the corticomedullary junction and outer medulla were scored by determining the percentage of damaged tubules that showed necrosis, cast formation, loss of brush border as follows: 0 = none, 1 =  < 10%, 2 = 11–25%, 3 = 26–45%, 4 = 46–75% and 5 =  > 76%^36^. At least 10 randomly selected fields from each sample were evaluated in a blind manner. Picrosirius red staining was performed by deparaffinizing and incubating a reaction solution prepared by dissolving Direct Red 80 in a saturated picric acid solution for 1 h. The quantification of picrosirius red positive areas were calculated from 7 randomly selected fields from each sample.

### Hydroxyproline assay

At day 14 after renal IR, the kidneys were removed from a mouse and homogenized by adding high-purity water. After centrifugation (4 °C, 5 min, 10,000 rpm), 10 N HCl was added to the pellets and the samples then incubated at 110 °C for 16 h, followed by being suspended in high-purity water. A chloramine-T solution (1.4% chloramine-T, 4.1% sodium acetate, 10% isopropyl alcohol) was added and the samples then incubated at room temperature for 20 min. Ehrlich’s reagent was added to the mixture which was then incubated at 65 °C for 15 min. The absorbance at 540 nm was then measured.

### Quantitative RT-PCR

Total RNA was extracted from 50–100 mg of kidney tissue at day 1 and day 14 after renal IR using RNAiso plus (Takara Bio, Japan). The RNA concentration was calculated from the absorbance at 260 nm, and cDNA was generated by reverse transcription with the Prime Script　RT master mix (Takara Bio, Tokyo, Japan). The PCR reaction was performed by mixing the Luna Universal qPCR Master Mix (New England Biolabs Japan) and the primers using the iCycler thermal cycler. The primers used for PCR are shown in Supplemental Table S1. GAPDH was used as an internal control.

### Purification of human AGP

Human AGP was purified as previously described^24,26^. Briefly, human plasma fraction V supernatant provided by KM Biologics Co., Ltd. (Kumamoto, Japan) was mixed with 10 mM acetate buffer and passed through a HiTrap CM FFcolumn (5 mL) and a HiTrap Q FF column (5 mL) on an AKTAprime Plus System (GE Healthcare, Tokyo, Japan). AGP was eluted with an acetate buffer containing 0.5 M NaCl and dialyzed against deionized water at 4 °C.

### Western blotting

Blood was collected before and at 8, 16, 24, 32, 40, 48, 56, 64, 72 h, 1, 7 and 14 days after renal IR. A 1 μL portion of plasma sample was loaded in 10% of SDS-PAGE gel. After running, it was transferred to an activated PVDF membrane at 100 V for 120 min and blocked with 5% milk at room temperature. After washing 3 times with 0.05% PBS-T, the primary antibody reaction with rabbit anti-ORM1 antibody (1:5000, Proteintech, IL) was incubated overnight at 4 °C. After washing, the secondary antibody reaction with goat anti-rabbit HRP antibody (1:5000) was incubated for 1 h at room temperature. ImageJ was used to quantify the signal intensity of the western blotting by reaction with HRP substrate (SuperSignal West Pico Plus chemiluminescent substrate, Thermo scientific, Waltham, MA).

### Cell cultures

RAW264.7 cells were obtained from the European Collection of Authenticated Cell Cultures, and cultured in DMEM medium containing 10% FBS, 100 U penicillin/mL and 100 mg streptomycin/mL at 37 °C in 5% CO_2_. atmosphere. Before the cells reach confluency, the adhered cells were detached using a cell scraper and seeded in a 6-well dish for use in in vitro experiments.

### Statistical analyses

The means for two group data were compared by the unpaired t-test. The means for the groups were compared by analysis of variance using Tukey’s multiple comparison method (using Graph Pad Prism 8, San Diego, CA). A probability value of *P* < 0.05 was considered to be significant.

## Supplementary Information

Supplementary Information
