# Supplementary material for: An acute phase protein α1-acid glycoprotein mitigates AKI and its progression to CKD through its anti-inflammatory action
Source: Sci Rep. 2021 Apr 12;11:7953. doi: 10.1038/s41598-021-87217-8 (PMC8041882; doi:10.1038/s41598-021-87217-8)
Supplement: Supplementary file 1 — Supplementary Information [file 41598_2021_87217_MOESM1_ESM.docx]

**An acute phase protein α_1_-acid glycoprotein mitigates AKI and its progression to CKD through its anti-inflammatory action**

Hiroshi Watanabe^1,*,#^, Rui Fujimura^1,2,*^, Yuto Hiramoto^1,*^, Ryota Murata^1^, Kento Nishida^1^, Jing Bi^1,2^, Tadashi Imafuku^1,2^, Hisakazu Komori^1^, Hitoshi Maeda^1^, Ayumi Mukunoki^3^, Toru Takeo^3^, Naomi Nakagata^3^, Motoko Tanaka^4^, Kazutaka Matsushita^4^, Masafumi Fukagawa^5^, Toru Maruyama^1^

^1^Department of Biopharmaceutics, Graduate School of Pharmaceutical Sciences, Kumamoto University, 5-1 Oe-Honmachi, Chuo-ku, Kumamoto 862-0973, Japan.

^2^Program for Leading Graduate Schools “HIGO (Health life science: Interdisciplinary and Glocal Oriented) Program”, Kumamoto University, 5-1 Oe-Honmachi, Chuo-ku, Kumamoto 862-0973, Japan

^3^Division of Reproductive Engineering, Center for Animal Resources and Development (CARD), Kumamoto University, Kumamoto, Japan

^4^Department of Nephrology, Akebono Clinic, 1-1 Shirafuji 5 Chome, Minami-ku, Kumamoto 861-4112, Japan

^5^Division of Nephrology, Endocrinology and Metabolism, Tokai University School of Medicine, 143 Shimo-Kasuya, Isehara, 259-1193, Japan.

^*^equal contribution: HW, RF and YH; ^#^Corresponding authors:

**Supplemental Materials**

**Figure S1. Systemic AGP knockout exacerbates renal injury at day 1 after renal ischemia-reperfusion.** (A) Body weight, (B) Kim-1 mRNA expression in the kidney, (C) representative photomicrographs of PAS-stained kidney sections at day 1 after renal IR are shown. Lower panels of PAS are an enlarged image of the upper panel. Original magnification: ×200 (upper panels), ×400 (lower panels). Scale bars represent 100 μm. Data are expressed as the mean±SE.

**Figure S2. Systemic AGP knockout exacerbates AKI to CKD progression.** (A) Blood urea nitrogen (BUN) and (B) serum creatine (SCr) were measured at day 14 after renal IR. hAGP administration to AGP KO mice had no effect on renal function. Data are expressed as the mean±SE.

**Figure S3.** **Post-administration of hAGP could not suppress renal inflammation and fibrosis at day 14.** (A) Schedule of post-administration of hAGP (2 mg/mouse/day, *ip*, from day 1 to day 7). (B) Blood urea nitrogen (BUN) was measured at day 1, 7, 14 after renal IR. (C) mRNA expression of α-SMA and collagen 1a2 in kidney and (D) Picrosirius red-stained kidney sections at day 14 after renal IR. Scale bars represent 100 μm. (E) mRNA expression of IL-6, TNF-α and IL-1β in kidney at day 14 after renal IR. Data are expressed as the mean±SE.

**Table S1**

The primers used for mRNA detection

| Target gene | Forward (5’→3’) | Reverse (5’→3’) |
| --- | --- | --- |
| Mouse |  | |
| GAPDH | AACTTTGGCATTGTGGAAGG | ACACATTGGGGGTAGGAACA |
| Kim-1 | TCCACACATGTACCAACATCAA | GTCACAGTGCCATTCCAGTC |
| IL-6 | TCTCTGCAAGAGACTTCCATCC | AGACAGGTCTGTTGGGAGTG |
| IL-1β | TGAGCTGAAAGCTCTCCACC | CTGATGTACCAGTTGGGGAA |
| TNF-α | CATGAGCACAGAAAGCATGATCCG | AAGCAGGAATGAGAAGAGGCTGAG |
| F4/80 | CATAAGCTGGGCAAGTGGTA | GGATGTACAGATGGGGGATG |
| Collagen 1a2 | CACCCCAGCGAAGAACTCATA | GCCACCATTGATAGTCTCTCCTAAC |
| α-SMA | AGCCATCTTTCATTGGGATGG | CCCCTGACAGGACGTTGTTA |
